# Typical versus delayed speech onset influences verbal reporting of autistic interests

**DOI:** 10.1186/s13229-017-0155-7

**Published:** 2017-07-21

**Authors:** Liliane Chiodo, Steve Majerus, Laurent Mottron

**Affiliations:** 1 Psychology & Neuroscience of Cognition Research Unit, PsyNCog University of Liège, Place des Orateurs, 1, Bâtiment 33, 4000 Liège, Belgium; 2 Centre d’Excellence en Troubles Envahissants du Développement de l’Université de Montréal, Hôpital Rivière-des-Prairies, 7070 Blvd Perras, Montréal, Québec H1E 1A4 Canada

**Keywords:** Autism spectrum, Speech onset delay, Restricted interests, Asperger syndrome, Heterogeneity

## Abstract

**Background:**

The distinction between autism and Asperger syndrome has been abandoned in the DSM-5. However, this clinical categorization largely overlaps with the presence or absence of a speech onset delay which is associated with clinical, cognitive, and neural differences. It is unknown whether these different speech development pathways and associated cognitive differences are involved in the heterogeneity of the restricted interests that characterize autistic adults.

**Method:**

This study tested the hypothesis that speech onset delay, or conversely, early mastery of speech, orients the nature and verbal reporting of adult autistic interests. The occurrence of a priori defined descriptors for *perceptual* and *thematic* dimensions were determined, as well as the perceived function and benefits, in the response of autistic people to a semi-structured interview on their intense interests. The number of words, grammatical categories, and proportion of *perceptual*/*thematic* descriptors were computed and compared between groups by variance analyses. The participants comprised 40 autistic adults grouped according to the presence (*N* = 20) or absence (*N* = 20) of speech onset delay, as well as 20 non-autistic adults, also with intense interests, matched for non-verbal intelligence using Raven’s Progressive Matrices.

**Results:**

The overall nature, function, and benefit of intense interests were similar across autistic subgroups, and between autistic and non-autistic groups. However, autistic participants with a history of speech onset delay used more perceptual than thematic descriptors when talking about their interests, whereas the opposite was true for autistic individuals without speech onset delay. This finding remained significant after controlling for linguistic differences observed between the two groups.

**Conclusions:**

Verbal reporting, but not the nature or positive function, of intense interests differed between adult autistic individuals depending on their speech acquisition history: oral reporting of intense interests was characterized by perceptual dominance for autistic individuals with delayed speech onset and thematic dominance for those without. This may contribute to the heterogeneous presentation observed among autistic adults of normal intelligence.

**Electronic supplementary material:**

The online version of this article (doi:10.1186/s13229-017-0155-7) contains supplementary material, which is available to authorized users.

## Background

Individuals on the autism spectrum (AS) are characterized by ‘highly restricted, fixated interests that are abnormal in intensity or focus (e.g., a strong attachment to, or preoccupation with unusual objects, excessively circumscribed or perseverative interests)’ according to the current DSM-5 AS criteria [1].^1^ The AS involves a large range of speech acquisition histories and verbal abilities which, along with developmental age, influence the expression of interests. At preschool age, intense interests of minimally verbal autistic children are directed towards specific perceptual properties of mostly inanimate objects, such as periodic movements or visual patterns [2], including letters and numbers [3–5]. As adults, 70 [6] to 90% [7] of verbal autistics have intense interests. Adult intense interests are characterized by the gathering of information on specific topics, resulting in encyclopedic knowledge. Intense interests are also known to persist across development [8]. Their relationship with other repetitive behaviors, as well as other behavioral characteristics, is poorly understood. Intense interests were initially described more frequently in adults with Asperger syndrome [9, 10] and autistic adults with high IQ [6], and the frequency is equivalent in these two DSM–IV clinical subgroups [8]. For non-autistic groups [11], intense interests have been reported to be more frequent in boys than girls [12] and in adult men than women [13].

Intense interests in AS individuals are not necessarily more frequent or more ‘restricted’ than in typical peers [11], but they differ in the level of accommodation required by families and by their inflexibility [7]. Topics of intense interests can be diverse: mechanics, language, mathematics, biology, taxonomies, and TV/videos [14]; Japanese comics, gadgets, dinosaurs, space/physics, natural disasters, power heroes, fact books, videogames, technical manuals, religion, politics, reptiles, and rodents [8]; facts/verbal or visual memory, classifying/ordering of information, dates and time, hoarding, and letters and numbers [2] are commonly observed topics.

The nature of the relationship between intense interests and other autistic behaviors, and recommendations on how to approach them, is poorly established. Intense interests may be relatively independent from other autistic repetitive behaviors [15, 16]. For some researchers, they have a detrimental effect on social development [8, 15] and should be suppressed. On the other hand, naturalistic behavioral intervention programs suggest integrating them into social routines [17–19]. More recent research indeed emphasizes their positive role in learning [20, 21], quality of life, and possibly language development [22, 23]. This positive view is also expressed by autistic adults when they describe their own intense interests [24–26].

Mechanistic accounts of intense interests were initially deficit-oriented. Following these accounts, intense interests result from a deficit in executive cognitive control [27, 28] or top-down processes [27, 28]. Indeed, neuroimaging studies show increased activity of the insula, a motivation-related neural region, and diminished activity in regions associated with cognitive control when autistic participants view objects associated with their domain of interest [29, 30]. However, these accounts lack empirical behavioral evidence, as there is currently no study that has shown an association between the level of inferred deficits in cognitive control and the frequency or the magnitude of intense interests.

Other accounts suggest that the domains of knowledge targeted by intense interests are those which ‘fit’ best autistic cognition. According to the hyper-systemizing model [31], the enhanced tendency to systemize in autistic individuals orients autistic people towards the detection and application of inflexible rules (if p, then q) mostly found in non-social information. The hyper-systemizing model can account for some broad domains of interests, such as physics or biology, which have been observed in autistic adults. Its explanatory power is less convincing for domains where rules are more arbitrary and unrelated, such as interests in written material in the case of ‘hyperlexic’ children; it also cannot account for the perceptual grounding of some intense interests, and their appearance in young children without oral speech. Alternatively, the veridical mapping [32] extension of the enhanced perceptual functioning model [33] grounds the nature and mechanisms of autistic intense interests on domain-specific expertise. This model proposes that perceptual expertise, mostly found in autistic people with speech onset delay, results from the superior performance, role, and autonomy of perception in autistic cognition. Conversely, speech-specific expertise is found in autistic people without speech onset delay (largely overlapping with the previous DSM-IV category of Asperger syndrome), accounting for enhanced regional cortical dedication of speech-related material in this autism subgroup [34]. This model accounts for the domain-specificity of intense interests, their behavioral and brain imaging correlates, the intrinsic association between intense interests and savant abilities, and the combination of interest and performance found in autistic individuals with limited speech. However, contrary to the hyper-systemizing account, this model poorly accounts for interests involving verbally expressed encyclopedic knowledge.

Here, we aimed to establish whether speech onset history may influence the nature and reporting of adult autistic interests by conducting a discourse analysis of autistic adults, with or without a history of speech onset delay, describing their interests during a semi-structured interview. Self-reports were analyzed for perceptual and thematic (i.e., those related to their semantic and conceptual dimension) content. Further questions of the interview assessed other aspects of the interests, such as their function, origin, emotional valence, and frequency. We predicted that autistic people without overt speech during preschool age would describe their interests focusing mostly on perceptual features related to the physical and surface properties of the surrounding world. In contrast, verbal reporting about their intense interests by autistic people who mastered speech at a typical age may be more strongly characterized by the thematic aspects of their interests.

## Methods

We conducted semi-structured interviews to assess whether speech acquisition history influences the nature of intense interests in AS. The following testing sequence was used: Anamnestic interview (age, sex, and level of education), the Autism Diagnostic Interview-Revised (ADI–R) [35], Raven Progressive Matrices, interests questionnaire, visuo-constructive and acoustic tasks (not presented here), the French version of the standardized Peabody Picture Vocabulary Test (PPVT), which evaluates receptive and expressive language (EVIP) [36], and a standardized reading task evaluating reading accuracy and speed reading test (Alouette) [37]. The participants’ responses to the interest questionnaire were digitally recorded then were analyzed based on lexical and grammatical content for the perceptual versus thematic nature of the descriptors used and the impact and function of the intense interests. The study was approved by the local ethics committee.

### Participants

Forty autistic adults (25 males, 15 females) between 18 and 41 years of age, diagnosed as autistic (5/40) or Asperger (35/40), according to DSM-IV criteria, by private psychiatrists or autism resource centers in Belgium, France, and Switzerland, were included in this study. The diagnosis and early speech history was obtained clinically and was validated by the ADI-R [35] already conducted with a parent for 17 of the participants. For the remaining 23 participants, we conducted the ADI-R with the parents (*N* = 20) or caregivers (*N* = 3) of the autistic participants to validate the clinical diagnosis. Autistic participants were allocated into two subgroups on the basis of having had (AS-SOD) or not (AS-NoSOD) a speech onset delay. This was documented by questions number 9 (one-word sentences) and 10 (two-word sentences) of the ADI-R. Speech acquisition was considered to be typical (*N* = 20) if single words were used before 24 months of age, and if two-word sentences were used before 33 months of age.

Twenty non-autistic adults (12 males, 8 females) between 18 and 41 years of age, without a history of psychiatric treatment or neurological disorders, were included in the control group of this study. These control participants, recruited via announcements of the study to personal and professional networks of the first author, dedicated more than 25% of their free time to an interest which was distinct from their professional activity. Their non-autistic status was verified by the administration of the ADI-R.

As shown in Table 1, the AS-SOD, AS-NoSOD and non-autistic control groups were matched for age, non-verbal intelligence using the Raven Progressive Matrices as well as Performance IQ measured with the Wechsler Adult Intelligence Scale–Forth Edition WAIS-IV [38]. However, there were significant between-group differences for Verbal IQ and Full-Scale IQ, receptive vocabulary measured by the EVIP test and education level. Also, the number of male participants was slightly larger in the AS-SOD group than in the other two groups, although this difference only reached significant when comparing the AS-SOD and AS-NoSOD groups. The ‘Alouette’ test ruled out any reading problems or dyslexia for all participants, and these test results will not be discussed further. None of the participants had an identified neuro-genetic condition based on their medical record.

**Table 1:** Characteristics of the AS-NoSOD, AS-SOD, and control groups

	AS-NoSOD	AS-SOD	Controls	AS-NoSOD/AS-SOD	AS-NoSOD/controls	AS-SOD/controls
				*p* values	*p* values	*p* values
Sample size (sex ratio)	20 (9 males, 11 females)	20 (16 males, 4 females)	20 (12 males, 8 females)	0.02	0.34	0.17
Age (SD)	29.65 (8.18)	26.3 (6.43)	27.25 (6.57)	0.3	0.29	0.67
Raven’s Progressive Matrices Raw scores (SD)	50.9 (8.14)	48.65 (8.04)	50.5 (7.06)	0.63	0.87	0.45
FSIQ (SD)^a^	119.25 (14.06)	88.5 (21.34)	111.9 (18.7)	<.001*	0.29	<.01*
VIQ (SD)^a^	129.06 (10.95)	92.75 (14.7)	114.36 (16.13)	<.001*	<.01*	<.001*
PIQ (SD)^a^	109 (15.93)	100.12 (24.97)	111.63 (20.12)	0.27	0.74	0.32
EVIP (SD)	127.1 (3.94)	111.75 (11.42)	122.9 (5.03)	<0.001*	0.08	<001*
ADI-R score:						
Social (SD)	20.15 (10)	21.35 (10)	1.15 (10)	0.38	<0.001*	<0.001*
Communic. (SD)	19.35 (8)	23.15 (8)	0.08 (8)	0.09	<0.001*	<0.001*
Interests (SD)	7.6 (3)	10.2 (3)	2.2 (3)	0.09	<0.01*	<0.001*
Age at first 2-word production (SD)	1.8 (0.44)	4.5 (1.75)	2 (0.39)	<0.001*	0.55	<0.001*
Level of education						
Post-secondary level	17	5	10	< 0.001*	0.01*	0.08
Secondary level	3	12	10	0.009*	0.02*	0.49
Special needs school	0	3	0	0.02*	1	0.07

*SD* standard deviation, *FSIQ* full-scale IQ (WAIS-IV), *VIQ* verbal IQ, *PIQ* performance IQ. ^a^This measure could be obtained for only a subset of participants (17 AS-NoSOD participants; 16 AS-SOD participants; 11 non-autistic control participants)

*EVIP* échelle de vocabulaire en image Peabody

*p < 0.05 for pairwise *t* test corrected for multiple comparisons, for all variables, except for level of education and gender ratio where χ^2^ tests were used

### Questionnaire for semi-structured interview

A verbal description of each participant’s intense interests was obtained through an oral questionnaire, inspired by the Yale survey of intense interests (as described in [39]), and a semi-structured interview developed by Mercier et al. [24]. The questionnaire contained 19 questions. Question 1 documented the intense interests of the participants by inviting them to describe his/her past and on-going interests. The question asked was: ‘Could you describe your past and present specific interests?’ The verbal reports obtained for this question were subjected to qualitative textual analysis to determine the perceptual versus thematic nature of the descriptors used by the participants when describing their intense interests (see below for a detailed description of the textual analysis). Questions 2 to 19 targeted the way intense interests are used in everyday life, as well as their perceived functions (origin of the interest, time taken by activities related to the interest, emotional valence of the interest, etc.). The verbal reports elicited by these questions were coded based on various response categories to enable between-group comparisons. The administration of the questionnaire took approximately 1 h and was performed at the participant’s home, at the university, or in a hotel. There was no time limit for the participants to respond.

### Analyses

We first conducted a lexical and grammatical analysis of the participants’ responses to characterize the linguistic properties of their answers for all questions; the amount of words produced was subjected to an ANOVA as a function of grammatical class and participant group. Next, we determined the perceptual or thematic nature of the intense interests based on the descriptors observed in the narrative for question 1; the proportion of descriptors was subjected to an ANOVA, as a function of the perceptual versus thematic nature of the descriptors and participant group. Last, we qualitatively assessed the impact and function of intense interests based on answers to questions 2–19; the responses to these questions were subjected to χ^2^ analyses.

### Lexical and syntactic analysis

A lexical and grammatical analysis of the participants’ verbal responses for questions 1 to 19 was performed using the automated text analysis software FrMG Wiki Alpage-Inria [40]. This software is based on a metagrammar which extracts a hierarchized tree of the syntactic structure of any sentence written in French. (examples of use: [41–43]). This allowed us to control for the possible influence of current differences in expressive language abilities in subsequent analysis of the reporting of intense interests. We extracted the number of words and grammatical classes for the ANOVA.

### Distinguishing the perceptual or thematic dimension of intense interests

The verbal content of the narratives for question 1 was analyzed to establish the proportion of perceptual versus thematic aspects of the participants’ answers. This was performed using the most widely used tool for qualitative textual analysis, NVivo 11 © [44]. This software detects, organizes, and analyses the content of verbal material, such as the percentage of verbal occurrences semantically related to target lexemes (minimal units of the lexicon, such as words) defined a priori by the experimenter. We therefore established an a priori list of lexemes semantically related to the concepts thematic and perceptual using official dictionaries of the French language. This list of semantically associated lexemes (55 for the thematic category and 72 for the perceptual category) was then validated by 2 professional linguists who were naïve to the purpose of the study. Validation consisted of removing lexemes that were judged to not be strongly related to the compound definitions of the concepts perceptual or thematic. Examples of lexemes related to the concept perceptual are aspect (appearance), couleur (color), détail (detail), lumière (light), ordre (order), and trait (line). Examples of lexemes related to the concept of thematic are analogie (analogy), connaissances (knowledge), relation (relation), and système (system). The final list of semantically associated lexemes is presented in supplementary material (see Additional file 1). These two lists of lexemes were then entered into NVivo 11 and their occurrence in the verbal reports of the participants determined. The lexemes preceding and following each occurrence of the target lexemes were also analyzed to clarify their meaning, if necessary. The proportion of *thematically* and *perceptually* related lexemes for each participant was determined by dividing the number of thematic/perceptual lexemes by the total number of words produced. These proportions were used for subsequent analysis (ANOVA).

### Impact and function of intense interests in daily life

The relative frequency of each category of response was calculated for each question, and the frequency distributions were compared across categories and groups using χ^2^ analysis.

## Results

### Lexical and syntactical analysis

We first analyzed the number of words for different grammatical categories (nouns, verbs, adjectives, adverbs, conjunctions, and pronouns) contained in the verbal reports in response to the various items of the questionnaire. A group by grammatical category ANOVA on the number of words indicated a main effect of group (F (2, 57) = 5.43, *p* < 0.01, η^2^_p_ = 0.16) and grammatical class (F (5, 285) = 25.73, *p* < 0.001, η^2^_p_ = 0.31), as well as a significant interaction (F (10, 285) = 4.70, *p* < 0.001, η^2^_p_ = 0.14). Newman-Keuls post hoc comparisons showed that, overall, the most frequently used grammatical class was verbs followed by nouns (both *p* < 0.05); the least frequently used grammatical class was adjectives. Although verbs were the most frequently used word type in the AS-NoSOD and non-autistic control groups, nouns and verbs were used at a similar frequency in the AS-SOD group. Otherwise, the three groups presented a similar distribution of responses as a function of grammatical class (see Table 2).

**Table 2:** Means and standard deviations for the number of words and grammatical category in the verbal reports to the interest questionnaire in function of group

Means	AS-NoSOD	AS-SOD	Controls	AS-NoSOD/AS-SOD	AS-NoSOD/controls	AS-SOD/controls
Nouns	242.30 (158.58)	129.35 (76.83)	550.20 (842.86)	.007*	.12	.03*
Verbs	406.60 (320)	133.80 (87.88)	745.45 (914.69)	<.001*	.13	.006*
Adjectives	90.60 (49.17)	35.05 (20.02)	146 (210.75)	<.001*	.26	.02*
Adverbs	229 (212.24)	73.75 (51.13)	439.45 (533.18)	.003*	.11	.004*
Conjunctions	148.45 (144.51)	39.70 (29.81)	291.15 (385.39)	.002*	.13	.006*
Pronouns	237.60 (179.71)	82.05 (56.87)	391.70 (445.49)	<.001*	.17	.004*

*p < .05 for Newman-Keuls post hoc comparisons

### Perceptual or thematic dimension of intense interests

Overall, the themes of the intense interests were similar across the three groups (see Additional file 2). The themes involved historical, technical, sporting, practical, literal, and social interests. A mixed ANOVA was performed with group as the between-subject factor, and the proportion of thematic/perceptual descriptors as the within-subject factor.

**Fig. 1:**
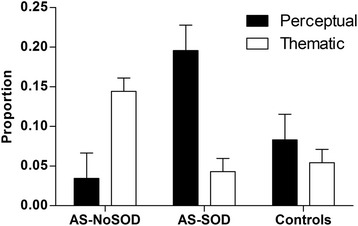
Proportion of thematic and perceptual descriptors used to describe intense interests, according to group

The main effects of group (F (2, 57) = 2.01, *p* = 0.14, η^2^_p_ = 0.07) and nature-of-interest (F (1, 57) = 1.27, *p* = 0.26, η^2^_p_ = 0.02) were not significant. However, the interaction was significant (F (2, 57) = 12.86, *p* < 0.001, η^2^_p_ = 0.31). Newman-Keuls post hoc tests showed that the AS-SOD group used more perceptual descriptors than both the AS-NoSOD (*p* < 0.001) and non-autistic control groups (*p* = 0.007), whereas the AS-NoSOD group used more thematic descriptors than both the AS-SOD (*p* = 0.03) and control groups (*p* = 0.04) (Fig. 1).

The group by nature-of-interest interaction remained significant when controlling for group differences in general verbal competency using receptive vocabulary (EVIP) (F (2, 56) = 5.16, *p* < 0.01, η^2^_p_ = 0 .16) or the total word count from the lexical and syntactic analysis, a proxy for grammatical complexity, as covariates (F (2, 56) = 11.95, *p* < 0.001, η^2^_p_ = 0.30).

Finally, we ran the same analyses, but limited the synonyms used to identify the interests to the nouns, to ensure that the observed group differences in lexical variety for the verbal reports did not bias the detection of the intense interests either in the perceptual or thematic dimension. This analysis led to similar results, showing a significant group by interest interaction (F (2, 57) = 11.27, *p* < 0.001, η^2^_p_ = 0.28). Newman-Keuls post hoc tests revealed that the AS-SOD group used more perceptual descriptors than both the AS-NoSOD (*p* < 0.001) and non-autistic control groups (*p* = < 0.001). Consistent with the previous analysis, only the interaction remained significant when controlling for group differences of receptive vocabulary (EVIP) (F (2, 56) = 3.53, *p* < 0.05, η^2^_p_ = 0.11), total word count (F (2, 5 6) = 10.13, *p* < 0.001, η^2^_p_ = 0.27), or total number of nouns produced (F (2, 56) = 10.63, *p* < 0.001, η^2^_p_ = 0.28).

Sex differences in interests have been reported [13]. Thus, we assessed the impact of sex differences by re-running the main mixed ANOVA on the proportion of thematic/perceptual descriptors with both participant groups and gender as between-subject factors. This analysis confirmed all previous effects while showing no evidence for an effect of gender, except for an interaction between gender and nature-of-interest. The results were: effect of group (F(2, 56) = 2.08, *p* = 0.13, η^2^_p_ = 0.07), effect of nature-of-interest (F(1, 56) = 2.67, *p* = 0.11, η^2^_p_ = 0.05), effect of gender (F(1, 56) = 0.21, *p* = 0.65, η^2^_p_ = 0.01), interaction between nature-of-interest and group, F (2, 56) =15.68, *p* < 0.001, η^2^_p_ = 0.36, interaction between nature-of-interest and gender, F (1, 56) = 4.35, *p* = 0.04, η^2^_p_ = 0.07. An exploration of this interaction by Newman-Keuls post hoc comparisons did not lead to any reliable differences, all *p* > .50 (mean proportions for male participants: perceptual descriptors = 0.10, thematic descriptors = 0.08; female participants: perceptual descriptors = 0.11, thematic descriptors = 0.08).

### Impact and function of intense interests in daily life

The comparison of the relative frequency of each category of response and the frequency distributions across categories and groups using χ^2^ analysis revealed no group differences for most of the answers to the 18 questions. This indicates that the overall impact and function of intense interests in the lives of the participants was largely similar across autistic subgroups and between the autistic and non-autistic groups (see Table 3). However, a few questions did reveal significant group effects. For question 2 (origin of intense interests), the AS-NoSOD group reported that a more general interest was at the basis of their intense interest more often than the other two groups; however, this difference was based on very few responses (four for the AS-NoSOD group, and zero for the other two groups) and this result must be viewed with caution. For question 6 (time spent on intense interests), the AS-NoSOD group spent more time on the intense interests than the other two groups, with the non-autistic control group spending the smallest amount of time. For question 13 (the pictorial versus textual nature of the interests), the non-autistic control group reported to have purely pictorial interests more often than the AS groups. Finally, for questions 14, 17, and 19 (classification of information, communication of information, and linking new information to existing knowledge), the AS-SOD group had many ‘do not know’ response codes, indicating that participants of this group had difficulties in responding to these questions.

**Table 3:** Response frequencies for questions 2–19 of the interests questionnaire, as a function of response code and participant group, with χ^2^ statistical values for the assessment of group effects

	AS-NoSOD	AS-SOD	Controls	χ^2^	*p*
2. From a historical perspective, what led you to get interested in your interests?					
Specific trigger	10	11	12	0.40	0.82
Global interest	4	0	0	8.57	0.01*
Social context	4	6	9	2.93	0.23
Do not know	4	3	2	0.78	0.68
3. Do you use your interests in everyday life?					
Yes	14	10	12	1.67	0.43
No	1	1	4	3.33	0.19
Do not know	5	9	4	3.33	0.19
4. Do your interests help you to have new ideas?					
Yes	14	10	14	2.30	0.32
No	2	1	2	0.44	0.80
Do not know	4	9	4	4.10	0.13
5. Do your interests help you to understand the things that surround you?					
Yes	10	9	15	4.21	0.12
No	1	4	1	3.33	0.19
Do not know	9	7	4	2.85	0.24
6. How much time do you spend on your interests?					
<25%	0	2	9	14.92	0.001*
25–75%	1	1	3	1.75	0.42
>75%	9	5	2	6.31	0.04*
Do not know	10	12	6	3.75	0.15
7. Do you talk about your interests with your family?					
Yes	14	8	13	4.25	0.12
No	3	6	4	1.37	0.50
Do not know	3	6	3	1.88	0.39
8. How do you feel about your interests?					
Positive emotion	7	8	11	1.76	0.41
Negative emotion	0	1	1	1.03	0.60
Impression of control	0	0	1	2.03	0.36
Impression of no control	3	0	1	3.75	0.15
Do not know	9	11	6	2.58	0.28
9. What are the positive aspects? °					
Yes	13	9	16	5.31	0.07
No	0	0	0	/	/
Do not know	7	11	4	5.31	0.07
10. What are the negative aspects?					
Yes	11	5	10	4.21	0.12
No	3	4	6	1.37	0.50
Do not know	6	11	4	5.71	0.06
11. Do you see a connection between these different interests?					
Yes	8	5	11	3.75	0.15
No	3	1	1	1.75	0.42
Do not know	9	14	8	4.14	0.13
12. By what means do you learn new elements related to your specific interests?					
Self-taught	10	8	12	1.6	0.45
Social context	2	0	3	3.05	0.22
Do not know	8	12	5	5.07	0.08
13. Are you interested in images or texts?					
Images	0	3	9	13.13	0.001*
Text	2	1	0	2.11	0.35
Images/text	9	5	5	2.46	0.29
Do not know	9	11	6	2.58	0.28
14. Do you classify information related to your interest, if so, how do you classify?					
Yes	7	3	7	2.63	0.27
No	3	3	8	4.66	0.10
Do not know	10	14	5	8.14	0.02*
15. Are you interested in some specific details or in all aspects of a piece of information?					
Detail	6	3	6	1.60	0.45
Global	3	1	4	2.02	0.36
Detail/global	2	3	3	0.29	0.87
Do not know	9	13	7	3.74	0.15
16. Do you think that this has an effect on your memory?					
Yes	9	7	8	0.42	0.81
No	1	2	4	2.26	0.32
Do not know	10	11	8	0.93	0.63
17. Can you explain what you have memorized to other people?					
Yes	9	5	8	1.87	0.39
No	3	0	4	4.20	0.12
Do not know	8	15	8	6.54	0.04*
18. Do you think that some knowledge is difficult to understand or to acquire?					
Yes	11	9	11	0.53	0.77
No	3	3	5	0.89	0.64
Do not know	6	8	4	1.90	0.39
19. When you learn new things, do you link these with what you already know?					
Yes	13	4	11	8.97	0.01*
No	0	0	2	4.14	0.13
Do not know	7	16	7	10.80	0.005*

*p < 0.05; note that due to the fact that not all participants reported positive/negative aspects, responses to these questions were coded as ‘yes’ when a positive/negative aspect was mentioned, as ‘no’ when no such aspect was mentioned, and as ‘do not know’ when participants could not answer to the question

## Discussion

This study represents the first qualitative investigation of intense interests in a large group of verbal AS adults by comparing autistic people with or without a history of speech onset delay. Furthermore, the interests in the AS groups were compared to those of non-autistic adults also showing intense interests. One important finding of this study is that the interests of autistic people with or without SOD were very similar in terms of topics, emotions produced, and adaptive benefits. However, although the *domains* of interests could be similar in AS subgroups (e.g., Harry Potter or Walt Disney World), the *vocabulary* used to describe them differed in terms of perceptual versus thematic descriptors.

### Benefit of interests

The interests were considered to have positive effects in both AS groups. The participants described their interests as being relevant to obtaining a job, increasing personal development, and understanding relations among people (AS-NoSOD), or preventing boredom, increasing intelligence, and resulting in respect from other people (AS-SOD). Initially, intense interests were considered negatively by experts [45, 46] and were suspected to have a detrimental effect on socialization, to prevent learning and to have no adaptive value [47], but see [24, 48, 49]. Attitudes and the judgment of non-autistic people on autistic intense interests, however, have recently evolved to converge with the findings of this study. For example, a study by Winter-Messiers [50] conducted on 23 AS-NoSOD individuals from 7 to 21 years of age revealed that intense interests improved self-esteem and quality of life, as indicated by emotional arousal when spending time on them. Improvement in speech quality, vocabulary, discourse organization, and transparence was observed when intense interests were evoked. Other benefits were fine motor skills, eye contact, initiation of conversation, and alleviating anxiety. Intense interests can also be used as motivational factors and to increase social relationships. Autistic children have also been shown to spend more time with other children who share their interests [17] and appear to improve their eye contact when doing so [51]. In summary, our results support the strength-based account of autistic intense interests described in recent literature [26].

### How speech acquisition history influences autistic interests

Our results indicate that the history of speech acquisition influences the report of intense interests. Autistic adults who had a history of speech onset delay report their interest using terms that predominantly refer to the perceptual dimension. In contrast, autistic adults with a typical speech acquisition history emphasize the thematic aspects of their interests, using terminology mostly related to the verbal expression of their semantic content. AS-SOD people used a priori defined perceptual descriptors more often than thematic ones, whereas the opposite was true for AS-NoSOD participants.

The question arises to what extent the perceptual versus thematic dominance of the descriptors for autistic interest reflects ‘deep’ differences between the nature of these interests. Both autistic subgroups were matched based on their score on the Raven Progressive Matrices. Thus, the limitation in verbal complexity evident in the AS-SOD group relative to the AS-NoSOD is unrelated to the complexity of the operations performed on the material of interest. Furthermore, the thematic versus perceptual dominance in the participants’ discourse, as well as for the grammatical complexity of language used, was independent of their current verbal knowledge as estimated by the EVIP.

In AS-SOD, the hypothesis of ‘visual thinking’, or a bias towards using visual representations, has been consistently confirmed at the cognitive level [52] and is plausibly related to ‘thinking in pictures’, as reported by some autistic adults [53]. Our findings provide a self-reported, measurable counterpart which supports this possible enhanced salience of perceptual dimension in otherwise similar domains of interests. Conversely, intense interests of AS-NoSOD people support the idea of an orientation towards the aspects that are more easily transferred in verbal code. The contrast in the way thematic or perceptual descriptors characterize the descriptors of interests used by AS-SOD and AS-NoSOD individuals suggests that autistic interests cannot be derived from early visual perceptual behaviors and orientation only, as initially suggested by our group [54]. Interests for visuo-perceptual dimensions are observed in a large subgroup of ‘prototypical’ autistic children with a history of SOD, and are still evident in adult autistic people. However, some AS individuals, with typical speech acquisition, have verbally oriented interests and this should be taken into consideration. An abstract (encompassing both verbal and non-verbal dimensions) model for the way intense interests develop in the AS should therefore be favored and include interests of autistic people not deprived of speech at an adult age.

### Hyper-systemizing or veridical mapping?

Various models for intense interests have unequal explanatory power. Some explain well early, perception-based interests manifested in preschool age children with speech onset delay, whereas others explain better adult, fluently verbal, ‘Asperger-type’ intense interests. The hyper-systemizing concept is orthogonal to this distinction. It has some explanatory power on the formal properties of the domains of interests, more evident when they are verbally expressed, but is not informative on the relationship between early domain-specific orientation (perception vs speech) and adult interests. According to the veridical mapping extension of the enhanced perceptual functioning model [32], the development of domain-specific expertise throughout life is one of the key factors of autistic phenotypic heterogeneity. In the AS-SOD group, enhanced top-down flow of perceptual information at early stages of autistic development orient them towards the detection of perceptual similarity. The initial over-development of ‘domain-specific’ pattern recognition and manipulation mechanisms, or perceptual expertise, orients autistic intelligence towards domains of knowledge highly loaded in structural analogy—while still retaining some aspects of their bottom up, perceptual origin and limiting their verbalization. In contrast, in the AS-NoSOD group, oral language would be the first and main investment, with a pervasive influence on the future extension and reporting of domains of interest. Autistic developmental pathways, strongly differing in their initial relationship with speech, converge towards similar interests, with similar benefits, but maintain subtle lifelong differences in their relationship with oral speech.

### Contribution to the definition of subgroups within the AS category

DSM-IV distinguished autism and Asperger syndrome according to the presence or absence of speech delay or abnormalities at a preschool age. However, the DSM-IV definition of Asperger syndrome was barely usable [55], of uncertain clinical value [56, 57], and did not allow clear-cut neurobiological distinctions [51, 58, 59]. It also resulted in including individuals presenting more autistic signs in the autistic group and individuals with superior estimated intelligence in the Asperger group [58]. The method used in the current study solves this problem by distinguishing non-verbal IQ-matched autistic individuals based on the history of speech acquisition during preschool years, and by further controlling for the impact of differences in verbal abilities on subsequent analyses, at the statistical level. The same strategy has been successfully used by others and revealed differences in AS people according to their history of speech development. For example, only AS-SOD individuals present greater perceptual capacities [60–63], whereas AS-NoSOD individuals present more motor difficulties [64]. Functional and structural differences at the neural level have also been observed in AS-SOD and AS-NoSOD groups, and are consistent with their cognitive differences. Autistic people with SOD show enhanced activity in perceptual expertise regions, whereas this is observed in language cortical regions in NoSOD individuals [65, 66]. They also show distinct patterns of brain volume difference in the corresponding regions [66]. Taken together, this suggests that having a history of speech onset delay, or not, may predict different brain structures and cognitive profiles, as well as behavioral phenotypes in adult autistics. The present study, showing that distinct characteristics define intense interests in AS-SOD and AS-NoSOD groups, provides further supportive evidence for the relevance of speech history in defining subgroups within the autism category.

### Limitations

This study has limitations. A written questionnaire and report may have reduced the difference in linguistic competence between the two autistic subgroups and produced different results. Autistic groups were not perfectly balanced for sex and the post hoc exploration of sex differences was insufficiently powered. Finally, the control for the effect of verbal intelligence was performed using a proxy for VIQ, the EVIP.

## Conclusions

Verbal reporting of intense interests of adult autistic people, with and without a history of speech onset delay, and a non-autistic control group, shows that the overall nature, function, and benefit of intense interests were similar across groups. However, autistic participants with a history of speech onset delay used more perceptual than thematic descriptors when talking about their interests, whereas the opposite was true for autistics without speech onset delay. This indicates that speech history influences the relevance of certain dimensions in the reporting of autistic interests, possibly based on contrasting early domain-specific expertise. Further investigations should be conducted using measures independent of current verbal ability to determine whether speech onset history actually orients not only the reporting, but also the content of autistic intense interests.

## Additional files

Additional file 1: List of semantically associated lexemes (55 for the thematic category and 72 for the perceptual category) used to identify thematic and perceptual descriptors in the verbal reports to question 1 of the interests questionnaire. (DOCX 16 kb)

Additional file 2: Schematic representation of the themes of intense interests observed in the AS-SOD, AS-NoSOD, and control groups. (DOCX 82 kb)

## Abbreviations

ADI-R: Autism Diagnostic Interview-Revised
ANOVA: Analysis of variance
AS: Autism spectrum
AS-NoSOD: Autism spectrum without delayed speech onset
AS-SOD: Autism spectrum with speech onset delay
EVIP: Échelle de vocabulaire en images Peabody
F: Female
IQ: Intelligence quotient
M: Male
WAIS: Wechsler Adult Intelligence Scale

## Footnotes

1 We will use the label ‘intense interest’ to refer to what has been classically designed as ‘circumscribed’ interests, and avoid offensive, deficit oriented language.
