# Supplementary material for: Typical versus delayed speech onset influences verbal reporting of autistic interests
Source: Mol Autism. 2017 Jul 21;8:35. doi: 10.1186/s13229-017-0155-7 (PMC5520365; doi:10.1186/s13229-017-0155-7)
Supplement: Supplementary file 1 — List of semantically associated lexemes (55 for the thematic category and 72 for the perceptual category) used to identify thematic and perceptual descriptors in the verbal reports to question 1 of the interests questionnaire. (DOCX 16 kb) [file 13229_2017_155_MOESM1_ESM.docx]

List of semantically associated lexemes for Thematic and Perceptual concepts

| Thematic synonyms  (55) | Perceptual synonyms  (72) |
| --- | --- |
| adaptations | alphabets |
| adaptation | alphabet |
| articles | watercolors |
| article | watercolor |
| analogies | aspects |
| analogy | aspect |
| analyses | calendars |
| analysis | calendar |
| trees | squares |
| tree | square |
| change | categories |
| changes | category |
| coherences | figures |
| coherence | figure |
| knowledge | collections |
| knowledges | collection |
| contexts | colors |
| context | color |
| cultures | dates |
| culture | date |
| discoveries | drawings |
| discovery | drawing |
| details | details |
| detail | detail |
| environments | aesthetics |
| environment | aesthetic |
| events | labels |
| event | label |
| evolutions | geometries |
| evolution | geometry |
| explanations | gestures |
| explanation | body language |
| functioning | hours |
| operation | hour |
| link | schedules |
| links | schedule |
| logic | images |
| logics | image |
| mechanisms | letters |
| mechanism | letter |
| organizations | lights |
| organization | light |
| origins | brands |
| origin | brand |
| course | models |
| reports | model |
| report | musics |
| relationships | music |
| relationship | shadows |
| strategies | shadow |
| strategy | orders |
| structures | order |
| structure | spelling |
| systems | spelling |
| system | photos |
|  | photo |
|  | pixels |
|  | pixel |
|  | portraits |
|  | portrait |
|  | tabeaux |
|  | table |
|  | features |
|  | line |
|  | types |
|  | type |
|  | visuals |
|  | visual |
|  | forms |
|  | form |
|  | characters |
|  | character |
