# Supplementary material for: Typical versus delayed speech onset influences verbal reporting of autistic interests
Source: Mol Autism. 2017 Jul 21;8:35. doi: 10.1186/s13229-017-0155-7 (PMC5520365; doi:10.1186/s13229-017-0155-7)
Supplement: Supplementary file 2 — Schematic representation of the themes of intense interests observed in the AS-SOD, AS-NoSOD, and control groups. (DOCX 82 kb) [file 13229_2017_155_MOESM2_ESM.docx]

Themes of intense interests

AS-NoSOD

Literal interests

Historical Interests

Social Interests

Sports and pratical interests

Technical/artistic Interests

Animals

Philosophy and words

Comics

Harry Potter

Fantastic universe

Magician tricks

Model airplanes

Zoology

Meteorolo-gy

Aviation

Movies and reviews

Nutrition therapy

Video games

Languages

Royalty

Figurines

Walt

Disney

Video games

Football

Equitation

Cooking

War

Themes of intense interests

AS-SOD

Technical/artistic Interests

Social Interests

Sports and practical interests

Literal interests

HistoricaI Interests

Dancing

Collages

Painting

Drawings

Photogra-phy

Harry Potter

Crystal

Video games

Automobi-les

Horses

Cooking

Medieval festivals

Role games

Languages

War

Aviation

Themes of intense interests

Controls

Technical/artistic interests

Social interests

Sports and pratical interests

Literal interests

HistoricaI interests

Fractals

Weapons

Aeronautics

Technolo-gy
